# The nuclear lamina is required for proper development and nuclear shape distortion in tomato

**DOI:** 10.1093/jxb/erad294

**Published:** 2023-07-28

**Authors:** Endia L Blunt, Junsik Choi, Hayley Sussman, Rachel C Christopherson, Patricia Keen, Maryam Rahmati Ishka, Linda Y Li, Joanna M Idrovo, Magdalena M Julkowska, Joyce Van Eck, Eric J Richards

**Affiliations:** The Boyce Thompson Institute, 533 Tower Road, Ithaca, NY 14853, USA; The Boyce Thompson Institute, 533 Tower Road, Ithaca, NY 14853, USA; The Boyce Thompson Institute, 533 Tower Road, Ithaca, NY 14853, USA; The Boyce Thompson Institute, 533 Tower Road, Ithaca, NY 14853, USA; The Boyce Thompson Institute, 533 Tower Road, Ithaca, NY 14853, USA; The Boyce Thompson Institute, 533 Tower Road, Ithaca, NY 14853, USA; The Boyce Thompson Institute, 533 Tower Road, Ithaca, NY 14853, USA; The Boyce Thompson Institute, 533 Tower Road, Ithaca, NY 14853, USA; The Boyce Thompson Institute, 533 Tower Road, Ithaca, NY 14853, USA; The Boyce Thompson Institute, 533 Tower Road, Ithaca, NY 14853, USA; The Boyce Thompson Institute, 533 Tower Road, Ithaca, NY 14853, USA; Cardiff University, UK

**Keywords:** Leaf morphology, nuclear lamina, nuclear shape, nuclear size, root growth, tomato

## Abstract

The nuclear lamina in plant cells is composed of plant-specific proteins, including nuclear matrix constituent proteins (NMCPs), which have been postulated to be functional analogs of lamin proteins that provide structural integrity to the organelle and help stabilize the three-dimensional organization of the genome. Using genomic editing, we generated alleles for the three genes encoding NMCPs in cultivated tomato (*Solanum lycopersicum*) to determine if the consequences of perturbing the nuclear lamina in this crop species were similar to or distinct from those observed in the model *Arabidopsis thaliana*. Loss of the sole NMCP2-class protein was lethal in tomato but is tolerated in Arabidopsis. Moreover, depletion of NMCP1-type nuclear lamina proteins leads to distinct developmental phenotypes in tomato, including leaf morphology defects and reduced root growth rate (in *nmcp1b* mutants), compared with cognate mutants in Arabidopsis. These findings suggest that the nuclear lamina interfaces with different developmental and signaling pathways in tomato compared with Arabidopsis. At the subcellular level, however, tomato *nmcp* mutants resembled their Arabidopsis counterparts in displaying smaller and more spherical nuclei in differentiated cells. This result argues that the plant nuclear lamina facilitates nuclear shape distortion in response to forces exerted on the organelle within the cell.

## Teaser

Altering the architecture of the nucleus, the cellular compartment that holds and organizes the genetic material, leads to changes in both nuclear shape and whole-plant development in tomato.

## Introduction

The functional consequences of altering the plant nuclear lamina have been explored most fully in *Arabidopsis thaliana* through ablation of genes encoding different nuclear lamina proteins, such as KAKU4 (Goto *et al.*, 2014) and members of the NMCP (nuclear matrix constituent protein)/CRWN (crowded nuclei) family (Masuda *et al.*, 1997; Dittmer *et al.*, 2007; Ciska *et al.*, 2013; Sakamoto and Takagi, 2013; Wang *et al*., 2013). These studies demonstrate the importance of the nuclear lamina in regulating nuclear shape and size. The loss of KAKU4 or a subset of CRWN/NMCP proteins in Arabidopsis leads to more uniform, spherical nuclei in enlarged, endoreduplicated cells that typically contain flattened or elongated nuclei in wild-type plants (Dittmer *et al.*, 2007; Sakamoto and Takagi, 2013; Wang *et al*., 2013; Goto *et al.*, 2014). This result runs counter to the situation in animal cells, in which disruption of the nuclear lamina typically results in misshapen nuclei with irregular margins (Mounkes *et al.*, 2003; Vergnes *et al.*, 2004; Scaffidi and Misteli, 2006; Gruenbaum and Medalia, 2015; de Leeuw *et al.*, 2018). Is the unexpected role of the nuclear lamina in specifying or stabilizing irregular nuclear shapes an idiosyncrasy of Arabidopsis or a more general property of plant nuclei?

NMCPs, the major protein component of the plant nuclear lamina, are partitioned into two clades: NMCP1-type and NMCP2-type, in angiosperms (Masuda *et al.*, 1997; Kimura *et al.*, 2010). In most flowering plant species, only a single NMCP2-type protein exists, accompanying one or more NMCP1-type paralogs (Poulet *et al.*, 2017). Loss of a full set of NMCP1-type proteins is lethal in Arabidopsis, indicating that the sole NMCP2-type protein, CRWN4, cannot cover all the essential functions provided by NMCP1-type proteins (Wang *et al*., 2013). Yet the converse is not true; the *CRWN4* gene is dispensable in Arabidopsis (Sakamoto and Takagi, 2013; Wang *et al*., 2013). Is this finding specific to Arabidopsis, or do NMCP2-type proteins in other angiosperms have unique functions that cannot be supplied by NMCP1-type proteins?

To address these questions, and to establish a new experimental system to investigate a fuller range of plant nuclear lamina function, we generated a complete set of *NMCP* gene mutations in cultivated tomato using CRISPR/Cas9 [clustered regularly interspaced short palindromic repeats (CRISPR)/CRISPR-associated protein 9] technology (Jinek *et al.*, 2012, 2013; Brooks *et al.*, 2014). Tomato is an agronomically important crop species that has a 7-fold larger and more complex genome (33% versus ~20% repetitive DNA), as well as a higher chromatin packing density, compared with Arabidopsis (Fujimoto *et al.*, 2005; Tomato Genome Consortium, 2012; Gaiero *et al.*, 2019; Wang *et al*., 2022). Therefore, tomato provides an experimental platform with a more typical angiosperm genome and nucleus to investigate the role of the plant nuclear lamina. Our results begin to differentiate between conserved functions of the nuclear lamina in angiosperms and those integrated into more specialized pathways that differ among taxa. Among the conserved functions is the facilitation or maintenance of non-spherical nuclear shapes in differentiated cells, as our findings in tomato mirror what we observed in Arabidopsis. Differences include the essential nature of the single NMCP2-type gene in tomato, arguing that NMCP2-type proteins, which form independent fibral networks in at least one angiosperm species (Masuda *et al.*, 2021), have essential functions distinct from their NMCP1-type counterparts. Our findings in tomato also underscore the diversity of whole-plant phenotypes that are dependent on the nuclear lamina.

## Materials and methods

### Plant materials and growth conditions

*Solanum lycopersicum* (tomato) cultivar M82 *SP*^+^ was used as the wild-type control and the background for the CRISPR/Cas9 transformants. Following *Agrobacterium tumefaciens*-mediated transformations (see below), well-rooted transformants were acclimated to greenhouse conditions as previously described (Brooks *et al.*, 2014; Van Eck *et al.*, 2019) with the following modifications: transformants were transplanted into the ‘Cornell’ soilless mix (1:1 mixture of sphagnum peat moss and vermiculite plus fertilizer and lime) supplemented with Osmocote Plus 15-9-12 slow release fertilizer (ICL), and then transferred to an environmental growth chamber for ~1 month under a 16 h light/8 h dark photoperiod at ~23 °C and 60% relative humidity. Plants were eventually transferred to a glasshouse under a 16 h light regime at 25–27 °C during the day and 17–18 °C at night. Subsequent generations were grown from seeds that were nicked with a razor blade to promote germination.

### CRISPR/Cas9 construct creation and *Agrobacterium tumefaciens*-mediated transformation

CRISPR/Cas9 constructs were designed using the online tool CRISPR-P 2.0 (Liu *et al.*, 2017). Two guide RNAs (gRNAs) were selected per gene to create deletions within their coding sequences. Target sequences were located within the first exon, when possible, and were separated by 40–72 bp. The primers used to amplify the gRNAs are listed in Supplementary Table S1 following the protocol described previously (Reem and Van Eck, 2019). Constructs were transformed into the tomato cultivar M82 *SP*^+^ using *A. tumefaciens* strain LBA4404 as previously described (Brooks *et al.*, 2014; Van Eck *et al.*, 2019); plant selection was carried out using kanamycin at a concentration of 200 mg l^–1^.

### Mutation detection and genetic analysis

Genomic DNA was extracted from 2–3 leaves per plant using the DNA extraction and purification protocol described by Cocciolone and Cone (1993). Plants were genotyped for the presence of Cas9 and indel polymorphisms at or near the gRNA target sites using PCR amplification from genomic DNA templates and oligonucleotide primers (see Supplementary Table S1). PCR products were resolved on either 1% or 2.5% (w/v) agarose gels, allowing larger deletion alleles to be detected and followed unambiguously. Small migration anomalies on higher percentage gels allowed us to detect even the small deletions present in the *nmcp2* alleles. The nucleotide sequence of the various alleles was determined using direct Sanger sequencing of amplification products utilizing genomic templates at the Cornell University Institute of Biotechnology Genomics Facility. We backcrossed all *nmcp* mutants three times to a recurrent wild-type M82 *SP*^+^ parent to generate isogenic mutant lines free of Cas9 and nptII (neomycin phosphotransferase II) transgenes. Our phenotypic analyses utilized backcrossed lines, with the exception of our imaging of guard cell nuclei for *nmcp1* mutants; we subsequently replicated and confirmed these nuclear morphology measurements using backcrossed material.

### cDNA analysis

To analyze the structure of the transcripts generated from the *nmcp1a-1* allele, we generated cDNA templates by first extracting RNA from a homozygous mutant plant using an E.Z.N.A. Plant RNA Kit (Omega Bio-tek). Following DNase I (New England BioLabs) treatment, cDNA was synthesized via the SuperScript IV First-Strand Synthesis System (Invitrogen). PCR amplification of *NMCP1A* transcripts was performed using gene-specific primers (see Supplementary Table S1); products were gel purified before performing independent Sanger sequencing reactions with either the forward or reverse amplification primers.

### Imaging nuclei

Leaf and root tissue were harvested and fixed in a 3:1 ethanol:acetic acid solution. Fixed tissue was rehydrated in water and stained using the fluorescent dye DAPI at a concentration of 2 µg ml^–1^. Samples were de-stained by incubation in water, mounted on a slide plus coverslip, and imaged using a Leica DM5500 epifluorescence microscope. For guard cell nuclei, images were analyzed using FIJI (https://fiji.sc/) software to generate nuclear size and roundness index measurements.

### Bioinformatics analysis

Phylogenetic tree analysis was done using the MEGA-11 package (https://www.megasoftware.net/) employing a maximum likelihood method (1000 bootstrap replicates) based on a MUSCLE-derived alignment of amino acid sequences corresponding only to the conserved coiled-coil region of the protein. The haplotype network shown in Supplementary Fig. S1 was generated manually based on Clustal Omega alignments performed on an EMBL-EBI web-based tool (https://www.ebi.ac.uk/Tools/msa/clustalo/) using sequence resources available through the Sol Genomics Network (https://solgenomics.net/); nucleotide sequences are provided in Supplementary Table S2. Coiled-coil analysis was done using the Waggawagga prediction package described by Simm *et al.* (2015) and available at https://waggawagga.motorprotein.de/.

### Root growth analysis

For our semi-automated analysis of root architecture, sterilized seeds were first germinated on medium containing 1/4 strength Murashige and Skoog (MS) (Murashige and Skoog, 1962) salts, MS vitamins medium, 0.5% (w/v) sucrose, 0.1% (w/v) MES, pH 5.8 (KOH), and 1% (w/v) agar. After 24 h of stratification at 4 °C in the dark to promote germination, the plates were maintained in a Conviron growth chamber with a light intensity of 130–150 µmol m^–2^ s ^–1^ in a 20 h light/4 h dark cycle at 26 °C and 60% humidity. For root growth assays, 4-day-old seedlings were transferred to freshly prepared 1/4 MS medium. Each plate (12 cm×12 cm, Greiner) contained one tomato seedling to enable a thorough evaluation of root system architecture. The plates were imaged daily for an additional 5 d using an Epson V800 scanner. To analyze root system architectural traits from the scanned plate images, a SmartRoot plugin (Lobet *et al.*, 2011) in ImageJ was used to trace the root manually, and extract root-related features in the CSV format, followed by data analysis in R.

## Results

**Fig. 1.**
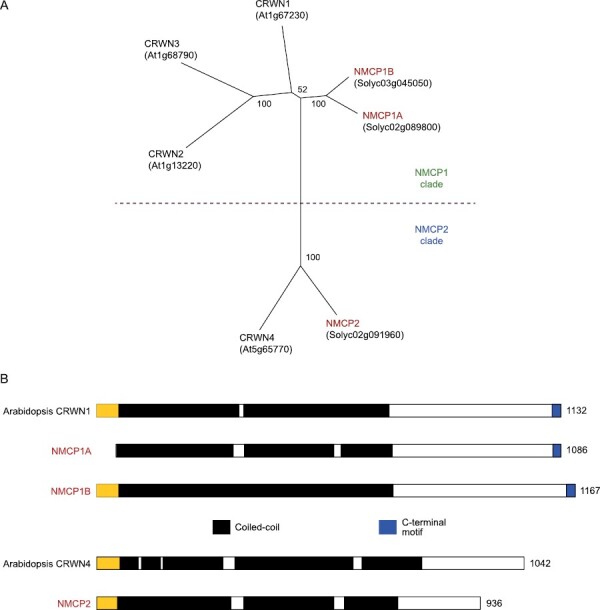
Comparison of NMCP proteins in tomato and Arabidopsis. (A) A distance tree constructed using an alignment limited to the conserved coiled-coil domains of CRWN/NMCP proteins in Arabidopsis and tomato (bootstrap values out of 100). Arabidopsis CRWN1, 2, and 3 are members of the NMCP1 clade, along with their tomato homologs, NMCP1A and NMCP1B. CRWN4 in Arabidopsis and its tomato ortholog, NMCP2, partition into the NMCP2 clade. (B) Domain structure of NMCP proteins in tomato with reference of Arabidopsis NMCP1-type (CRWN1) and NMCP2-type (CRWN4) proteins. Numbers refer to amino acid coordinates. The size and position of coiled-coil regions are shown in black, while a conserved C-terminal motif is denoted in blue. The N-terminal region, which is missing in NMCP1A from tomato, is designated in yellow.

The tomato genome contains three genes encoding NMCP proteins, comprising two NMCP1-type proteins [hereafter called NMCP1A (Solyc02g089800) and 1B (Solyc03g045050)] and a single NMCP2-type protein, designated NMCP2 (Solyc02g091960) (Fig. 1A). The structures of the predicted proteins conform to the expectation for NMCPs; each includes a coiled-coil domain of several hundred amino acids, followed by a C-terminal region (blue region in Fig. 1B). The extreme C-terminus of NMCP1A and NMCP1B contains a short (~15 amino acids) conserved motif, which is implicated in abscisic acid (ABA) regulation in Arabidopsis (Zhao *et al.*, 2016). This motif is absent in NMCP2, as is typical for dicot NMCP2-type proteins (Wang *et al*., 2013). Both NMCP1B and NMCP2 have a short N-terminal domain (yellow region in Fig. 1B), completing the prototypic tripartite structure of NMCPs (Ciska *et al.*, 2013), but NMCP1A is unusual in the absence of this N-terminal domain. Interestingly, NMCP1A orthologs in some but not all closely related *Solanum* species do contain an N-terminal domain. The haplotype network shown in Supplementary Fig. S1 indicates that this domain was lost independently at least three times since the divergence of *Solanum* species [the potato–tomato lineages split an estimated 8 million years ago (Särkinen *et al.*, 2013)], and occurred prior to the domestication of cultivated tomato.

There is no indication that *NMCP1A* in tomato is a pseudogene as transcripts of the gene are present in all tissues, based on public transcription profiling data (see Supplementary Fig. S2) (Zouine *et al.*, 2017). Indeed, all three *NMCP* genes are broadly expressed, with higher transcript abundance in proliferating tissues, such as shoot apical meristems and leaf primordia (Supplementary Fig. S2) *NMCP1B* transcripts tend to be more abundant in comparison with transcripts of its paralog, *NMCP1A*, by a factor of 2- to 3-fold.

### Generating mutations in the three *Solanum lycopersicum NMCP* genes

We generated mutations in each of the three *NMCP* genes using CRISPR/Cas9 technology (Brooks *et al.*, 2014). We designed constructs to express the Cas9 endonuclease along with two gRNA targeting sites within or near the first coding exon of each gene. We selected gRNA sites spaced a few dozen base pairs apart to facilitate recovery of larger deletions more likely to disrupt gene function (Supplementary Fig. S3). These constructs were introduced into the M82 *SP*^+^ cultivar using *Agrobacterium*-mediated transformation (Van Eck *et al.*, 2019), and transgenic lines were screened for mutations at the targeted loci. As shown in Supplementary Fig. S3 and summarized in Table 1, we recovered two complex deletion mutations each for the *NMCP1A* and *NMCP1B* genes.

**Table 1.** CRISPR/Cas9-derived alleles in cultivated tomato (*Solanum lycopersicum*) cultivar M82 *SP*^+^

Gene	Allele	Nucleotide change	Effect on coding sequence
*NMCP1A*	*nmcp1a-1*	97 bp deletion plus 4 bp insertion spanning exon 1 and intron 1	Aberrant splicing, frameshift, and premature termination
	*nmcp1a-2*	66 bp deletion spanning exon 1 and intron 1	Premature termination after nine amino acids
*NMCP1B*	*nmcp1b-1*	4 bp deletion in exon 1, plus 128 bp deletion spanning exon 1 and intron 1	Frameshift in exon 1
	*nmcp1b-2*	2 bp deletion in exon 1, plus 218 bp deletion plus 9 bp insertion spanning exon 1 and intron 1	Premature termination after 25 amino acids
*NMCP2*	*nmcp2-1*	4 bp deletion in exon 1	Frameshift and premature termination in exon 1
	*nmcp2-2*	4 bp deletion in exon 1	Frameshift and premature termination in exon 1
	*nmcp2-3*	Two 3 bp deletions in exon 1	Amino acid substitution F44Y plus deletions of residues D45 and L64

Based on their size and structure, we predict that these *nmcp1a* and *nmcp1b* mutations should destroy, or severely compromise, gene function. The *nmcp1a-1* deletion clips the first exon within the sixth codon and extends into the first intron; cDNA analysis indicated that this allele leads to use of an alternative splice donor site and addition of 28 amino acids derived from an out-of-frame translation of exon 2 sequences (Supplementary Fig. S3A). The other *nmcp1a* allele (*nmcp1a-2*) is a deletion that truncates the first exon within the eighth codon and leads to a predicted premature stop after only nine amino acids, with the terminal codons derived from intron 2 sequences (Supplementary Fig. S3A). Of the two *nmcp1b* alleles recovered, *nmcp1b-2* is the best candidate for a null mutation as it contains a 2 bp deletion at the promoter-proximal gRNA target site that leads to two closely spaced stop codons after an ORF encoding only 25 amino acids (Supplementary Fig. S3B). The *nmcp1b-1* allele contains a 4 bp deletion at the same gRNA target site that shifts the translational reading frame after only 25 amino acids, as well as a larger deletion spanning the distal end of exon 1 and a portion of intron 1 (Supplementary Fig. S3B). Use of alternative splice donor sites in *nmcp1b-1* transcripts, however, could lead to translation of downstream exons in the proper reading frame. Consequently, much of our phenotypic analysis relied on the use of the candidate null allele, *nmcp1b-2*.

For *NMCP2*, we recovered two independent 4 bp deletions (*nmcp2-1* and *nmcp2-2*) that cause a translational frameshift after codon 43, each leading to a 16 amino acid out-of-frame translation and premature chain termination within the first exon (Supplementary Fig. S3C; Table 1). Therefore, we predict that both *nmcp2-1* and *nmcp2-2* are null alleles. In addition, we recovered a more complex allele that contains two 3 bp deletions, one at each of the two gRNA target sites, creating the *nmcp2-3* allele that leads to one amino acid substitution and two single amino acid deletions in the boundary between the short N-terminal domain and the start of the conserved coiled-coil region (Supplementary Figs S3C, S4A). Using this set of mutations, we began to explore the consequences of perturbation of the nuclear lamina at both the whole-plant and organelle level.

### Loss of *NMCP1B* leads to developmental abnormalities

**Fig. 2.**
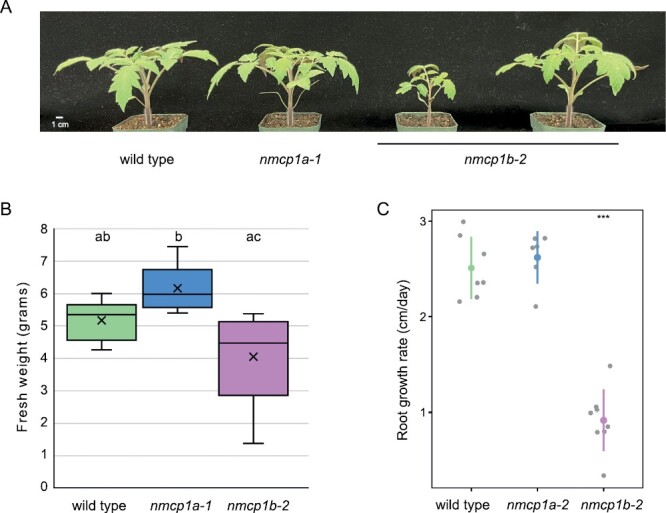
Growth characteristics of *nmcpa1* and *nmcp1b* mutants. (A) Images of the shoots of a wild-type M82 individual, a representative *nmcp1a-1* homozygote, and two sibling *nmcp1b-2* homozygotes; all plants were grown in parallel and are 23 d old. Scale bar, 1 cm. (B) Shoot fresh weight of M82 wild-type individuals (*n*=9) compared with *nmcp1a-1* homozygotes (*n*=8) and *nmcp1b-2* homozygotes (*n*=9); all plants were grown in parallel and were 24 d old when shoot tissue was harvested. Samples that share a letter designation (a, b, c) were not statistically different based on one-way ANOVA with post-hoc Tukey HSD tests. (C) Main root growth rate of wild-type M82 (2.5 ± 0.3 cm d^–1^, *n*=7), *nmcp1a-2* homozygotes (2.6 ± 0.3 cm d^–1^, *n*=6), and *nmcp1b-2* homozygotes (0.92 ± 0.3 cm d^–1^, *n*=8). Student’s *t*-test for each mutant compared with the wild type; ***P<0.001 for the wild type versus *nmcp1b-2*, while the *nmcp1a-2* genotype was not significantly different from the wild type.

We began our phenotypic analysis with mutants containing disruptions of either *NMCP1A* or *NMCP1B*, the two paralogous *NMCP1*-type genes in tomato. Homozygotes for the *nmcp1a* or *nmcp1b* alleles were viable. While *nmcp1a* mutants were fertile and showed no obvious whole-plant defects, *nmcp1b-2* mutants exhibited a suite of phenotypes. The most obvious trait was reduced plant size, especially during the early growth phase, but this phenotype displayed variable expressivity. Figure 2A illustrates this variability in shoot growth; some *nmcp1b-2* individuals in a family derived from a backcrossed homozygote displayed near-normal stature, while other siblings were smaller. As quantified in Fig. 2B, the average fresh weight of *nmcp1a* plants (shoot tissue) was comparable with that of a matched wild-type cohort, but the fresh weight of *nmcp1b* individuals trended lower and exhibited more variation. We observed a >2-fold reduction in the average growth rate of primary roots from *nmcp1b* seedlings (0.92 ± 0.3 cm d^–1^) compared with the wild type (2.5 ± 0.3 cm d^–1^), while the growth rate for *nmcp1a* seedling primary roots was unaffected (2.6 ± 0.3 cm d^–1^) (Fig. 2C). These observations indicate that loss of the most highly expressed *NMCP1*-type gene in tomato, *NMCP1B* (Zouine *et al.*, 2017), leads to reduced vigor, while loss of *NMCP1A* does not cause significant effects at the whole-plant level, with one notable exception. We observed a characteristic abnormal leaf morphology phenotype with incomplete penetrance in both *nmcp1a* and *nmcp1b* mutants. As shown in Supplementary Fig. S5, affected plants exhibited a folded terminal leaflet frequently accompanying a marked leaf asymmetry. Approximately 10–20% of the *nmcp1a* or *nmcp1b* mutant individuals in a typical true-breeding family exhibited terminal leaf folding. When this phenotype appeared, it was restricted to the first (or, less frequently, the second) leaf to emerge from the meristem.

### Abnormal nuclear morphology in *nmcp1a* and *nmcp1b* mutants

**Fig. 3.**
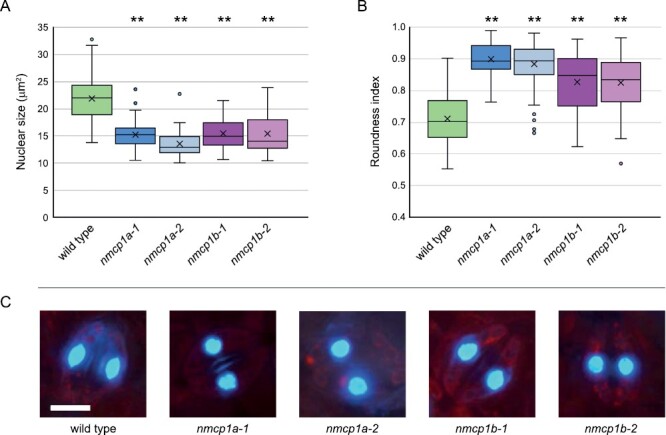
Mutations in the *NMCP1* genes reduce nuclear size and alter nuclear shape in guard cells. We imaged DAPI-stained nuclei from guard cells on the abaxial (lower) surface of the terminal leaflet of the third leaf of 5- to 7-week-old plants of the M82 wild type and homozygotes for the indicated alleles of either *NMCP1* paralog. (A and B) Box-and-whisker plots displaying nuclear size (A), as reflected by cross-sectional area, or nuclear shape (B), where the roundness index equals the inverse of the aspect ratio (long/short axis). The numbers of nuclei measured: wild type (*n*=71), *nmcp1a-1* (*n*=81), *nmcp1a-2* (*n*=81), *nmcp1b-1* (*n*=74), and *nmcp1b-2* (*n*=76). **P<0.01 based on a one-way ANOVA with post-hoc Tukey HSD test for each genotype in comparison with the wild-type M82 sample. (C) Representative DAPI-stained guard cell nuclei from the different genotypes (scale bar, 10 μm). Note that leaf guard cells in cultivated tomato are 2*n* (Jacobs and Yoder, 1989); therefore, endopolyploidy is not a complicating factor in this analysis.

Moving to the subcellular level, we examined nuclear phenotypes caused by the loss of *NMCP1A* or *NMCP1B*. We first imaged nuclei in a distinct cell type to make a more direct comparison among genotypes and to avoid cell type-specific differences in nuclear morphology. We focused on guard cells on the abaxial surface of leaves, which are consistently sized and diploid (Sax and Sax, 1937; Tan and Dunn, 1973; Jacobs and Yoder, 1989). As shown in Fig. 3, wild-type leaf guard cells typically had lens-shaped nuclei with a roundness index of 0.71 ± 0.08 [the inverse of the aspect ratio (long/short axis) of ~1.4]. In comparison, leaf guard cell nuclei in mutants lacking either *NMCP1A* or *NMCP1B* were more spherically shaped, displaying a roundness index closer to 1; for *nmcp1a* samples, 0.9 ± 0.07, and for *nmcp1b* mutants, 0.83 ± 0.09. Further, *nmcp1a* and *nmcp1b* guard cell nuclei were smaller than wild-type nuclei as reflected in a reduction in cross-sectional area; wild type, 22 ± 4.0 µm^2^ compared with ~15 ± 3.0 µm^2^. These results demonstrate that inactivation of either *NMCP1*-type gene in tomato leads to smaller and more spherically shaped nuclei in leaf guard cells.

Our finding that mutants defective in either NMCP1-type paralog exhibited similar nuclear phenotypes in guard cells indicates that both NMCP1A and NMCP1B are required to specify and/or maintain proper nuclear morphology. Even though NMCP1A lacks an N-terminal domain and is expressed at a lower level than NMCP1B (Supplementary Fig. S2) (Zouine *et al.*, 2017), loss of *NMCP1A* led to a nuclear phenotype of equivalent severity to that seen in *nmcp1b* mutants. Therefore, the more highly expressed NMCP1B paralog with a canonical three-domain structure is not able to fulfill the functional requirement for the NMCP1A paralog, at least in certain cell types.

Next, we examined nuclei in root epidermal cells. In contrast to the guard cells, root epidermal cells are enlarged and often endopolyploid in the maturation zone (Smulders *et al.*, 1994). Consequently, these cells contain larger nuclei compared with diploid cells and, in some cases, the nuclei are elongated significantly. In wild-type roots, we observed epidermal root cells with dramatically elongated nuclei, as illustrated in Supplementary Fig. S6. Elongated nuclei were also found in root epidermal cells in *nmcp1a* mutants (Supplementary Fig. S6A), suggesting that the loss of NMCP1A function has less or no effect in root tissue. In contrast, nuclei in root epidermal cells from *nmcp1b* mutants were more spherical in shape relative to the wild type (Supplementary Fig. S6B), consistent with the effect we observed in guard cells. Taken together, these observations indicate that the two tomato NMCP1 proteins are necessary to regulate or facilitate the adoption of non-spherical nuclear shapes in guard cells, but that nuclear elongation in other differentiated cell types, such as root epidermal cells, might rely primarily on NMCP1B.

### Functional relationship between NMCP1A and NMCP1B

**Fig. 4.**
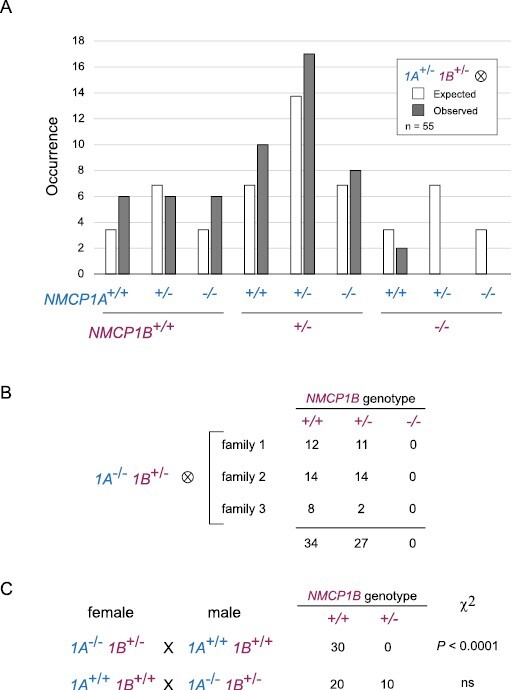
Genetic behavior of *nmcp1a* and *nmcp1b* loss-of-function mutations. (A) Transmission of *nmcp1a* and *nmcp1b* mutations in a segregating F_2_ population. (B) Segregation of an *nmcp1b* mutation in the absence of *NMCP1A* function after self-pollination of *1A*^*–/–*^*1B*^*+/–*^ parents. (C) Results of reciprocal crosses between wild-type individuals and *1A*^*–/–*^*1B*^*+/–*^ individuals to examine the co-transmission of the *nmcp1a* and *nmcp1b* alleles through either the male or female gametophyte. ns, not significant.

We determined whether it was possible to recover plants that completely lacked NMCP1-type function. We crossed *nmcp1a-1* and *nmcp1b-2* homozygotes to generate a trans­heterozygote F_1_ parent, which was then self-pollinated. In the F_2_ generation, we recovered *nmcp1a-1/nmcp1a-1; NMCP1B/nmcp1b-2* plants (*1A*^*–/–*^*1B*^*+/–*^ for simplicity), indicating that a single wild-type *NMCP1B* allele, in the absence of NMCP1A function, is sufficient for viability in a diploid sporophyte. However, no *1A*^*+/–*^*1B*^*–/–*^ or *1A*^*–/–*^*1B*^*–/–*^ individuals were observed in the F_2_ generation (Fig. 4A).

The inability to recover *1A*^*–/–*^*1B*^*–/–*^ plants from this cross could result from two different causes: (i) lethality of the double null combination in the diploid sporophyte; or (ii) lethality of haploid *nmcp1a nmcp1b* gametophytes from one direction of the cross. We began to distinguish between these possibilities by examining the selfed progeny of *1A*^*–/–*^*1B*^*+/–*^ parents. Among the progeny, we recovered only *1A*^*–/–*^*1B*^*+/+*^ and *1A*^*–/–*^*1B*^*+/–*^ individuals in a ratio consistent with a 1:1 segregation (Fig. 4B). These results are most easily explained by the inviability of either male or female *nmcp1a nmcp1b* gametophytes. We tested this prediction by performing reciprocal crosses between wild-type and *1A*^*–/–*^*1B*^*+/–*^ plants. We found that only *nmcp1a NMCP1B* gametophytes were transmitted through the pistillate parent, while both possible genotypes were transmitted through the pollen (Fig. 4C). These results indicate that *nmcp1a nmcp1b* female gametophytes are inviable, demonstrating that the loss of both NMCP1-type proteins leads to lethality in the female haploid lineage.

**Fig. 5.**
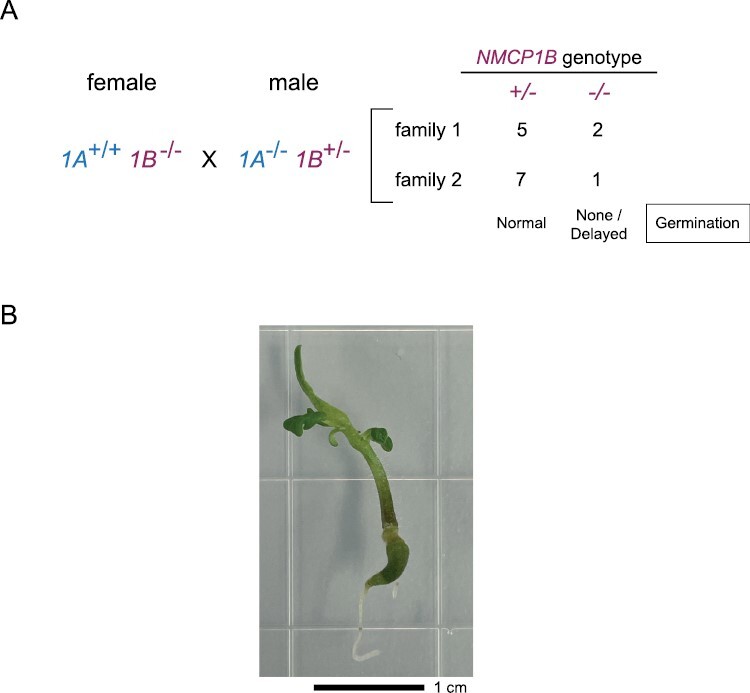
Developmental defects in seedlings carrying a single *NMCP1A* allele in the absence of *NMCP1B* function. (A) Crossing scheme to recover *1A*^*+/–*^*1B*^*–/–*^ seeds, which show no or delayed germination. (B) Morphology of a *1A*^*+/–*^*1B*^*–/–*^ seedling (14 d old) dissected from a late germinating seed and subsequently grown on solid agar medium supplemented with sucrose; note abnormal cotyledon development, the swollen green primary root axis, and delayed rootlet growth.

The inviability of *nmcp1a nmcp1b* female gametophytes explains the lack of *1A*^*–/–*^*1B*^*–/–*^ plants among the selfed progeny of *1A*^*+/–*^*1B*^*+/–*^ parents. Yet, we recovered *1A*^*–/–*^*1B*^*+/–*^ F_2_ segregants at close to the expected frequency (Fig. 4A), and therefore we anticipated that *1A*^*+/–*^*1B*^*–/–*^ individuals would be found as well. The absence of *1A*^*+/–*^*1B*^*–/–*^ segregants might be due to the haplo-insufficiency of a single *NMCP1A* allele in the absence of *NMCP1B* function. To increase our chances of recovering *1A*^*+/–*^*1B*^*–/–*^ individuals, we crossed *1A*^*+/+*^*1B*^*–/–*^ plants as females with pollen from *1A*^*–/–*^*1B*^*+/–*^ plants, because we knew that it was possible to transmit the *nmcp1a-1 nmcp1b-2* combination through the male gametophyte. When seeds from this cross were planted in soil, only *1A*^*+/–*^*1B*^*+/–*^ F_1_ individuals were recovered. However, the germination rate was lower than expected. We therefore plated seeds from *1A*^*+/+*^*1B*^*–/–*^×*1A*^*–/–*^*1B*^*+/–*^ crosses on MS medium plates supplemented with sucrose to either observe or recover defective embryos or seedlings from any non-germinating seeds. As shown in Fig. 5A, we examined two families from this cross and found that germinating seeds gave rise to *1A*^*+/–*^*1B*^*+/–*^ seedlings. The few non-germinating seeds present in these families were dissected to recover embryos, which had the missing genotype *1A*^*+/–*^*1B*^*–/–*^. In a few cases, we were successful in removing the seed coat to facilitate a delayed germination, leading to recovery of *1A*^*+/–*^*1B*^*–/–*^ seedlings. These individuals grew slowly on the agar plates and exhibited a range of developmental defects, including an enlarged, green primary root axis and a disorganized shoot apex that produced small leaf-like structures (Fig. 5B). These *1A*^*+/–*^*1B*^*–/–*^ seedlings survived transplantation to soil and grew slowly, with the first emerging leaf-like structures showing asymmetry, and, in one case, a trumpet shape (Supplementary Fig. S7A). Similar to the situation for single *nmcp1a* or *ncmp1b* mutants, later emerging leaves had flatter, more symmetrical leaflet shapes. These *1A*^*+/–*^*1B*^*–/–*^ individuals were severely dwarfed (Supplementary Fig. S7B), with adult leaves that were slightly smaller and darker in color (Supplementary Fig. S7C), but eventually produced flowers with morphologies resembling the wild type.

These results demonstrate that eliminating NMCP1 activity leads to gametophytic lethality in the female lineage and that a severe reduction in NMCP1 activity leads to developmental defects in the diploid sporophyte. It is likely that a sporophyte completely missing NMCP1 function would not survive, but the inviability of *nmcp1a nmcp1b* female gametophytes makes it impossible to construct the double null *1A*^*–/–*^*1B*^*–/–*^ genotype through traditional crosses. Our genetic findings also shed light on the functional differences between *NMCP1A* and *NMCP1B*. Plants with the *1A*^*–/–*^*1B*^*+/–*^ genotype grew to a normal stature with no gross developmental abnormalities, while *1A*^*+/–*^*1B*^*–/–*^ individuals grew slowly, were severely dwarfed, and showed altered morphology in the first emerging leaves. These observations demonstrate that a single *NMCP1A* allele is less able to support essential functions compared with a single functional copy of *NMCP1B*.

### Loss of *NMCP2* leads to sporophytic lethality

We next turned our attention to the investigation of the role of NMCP2. As shown in Table 1, we isolated three alleles of *NMCP2*, including two predicted null alleles, each in the form of a 4 bp deletion frameshift mutation in the first coding exon. Attempts to recover plants that were homozygous for either of these alleles (*nmcp2-1* and *nmcp2-2*) were unsuccessful. Self-pollinating heterozygotes carrying either suspected *nmcp2* null mutation yielded only wild-type and heterozygote individuals in a ratio consistent with 1:2:0 Mendelian segregation (for *nmcp2-1* heterozygotes: +/+:+/–:–/– :: 10:20:0; for *nmcp2-2* heterozygotes: +/+:+/–:–/– :: 9:16:0); both results are incompatible with a standard 1:2:1 Mendelian segregation ratio, *P*<0.02. Reciprocal crosses between *nmcp2-1* heterozygotes and wild-type plants indicated that both female and male *nmcp2* gametophytes are viable, without significant deviations from the expected 1:1 segregation ratio (+/+:+/– :: 9:5 when the heterozygote was the female parent, *P* ~0.29; and +/+:+/– :: 11:13 when the heterozygote was the pollen donor, *P* ~0.68). These data indicate that *nmcp2* null alleles are sporophytic lethal mutations and that NMCP2 function is required for the viability of the diploid plant.

### Alterations near the N-terminal domain of *NMCP2* cause defects in nuclear morphology

**Fig. 6.**
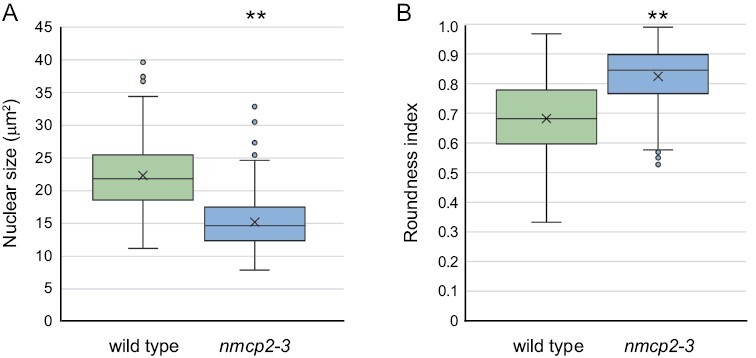
Alteration in the N-terminal domain of NMCP2 reduces nuclear size and changes the nuclear shape in guard cells. We imaged DAPI-stained nuclei from guard cells on the abaxial (lower) surface of the terminal leaflet of developmentally matched leaves from 3-week-old plants of the M82 wild type and *nmcp2-3* homozygotes. (A and B) Box-and-whisker plots displaying nuclear size (A), as reflected by cross-sectional area, or nuclear shape (B), where the roundness index equals the inverse of the aspect ratio (long/short axis). The number of nuclei measured is 186 for each sample, which was derived from two biological replicates. **P<0.01 based on a one-way ANOVA with post-hoc Tukey HSD test.

In contrast to the situation for the *nmcp2* null alleles, we recovered homozygotes carrying the *nmcp2-3* allele, which encodes a protein containing three amino acid alterations, including an amino acid substitution (F44Y) and an amino acid deletion (D45) within the region adjacent to the N-terminal domain that is predicted to form an extended coiled-coil domain in NMCP2-type proteins (Table 1; Supplementary Fig. S4A). The third change caused by the *nmcp2-3* allele is a deletion of a well-conserved lysine residue (L64) at the start of the coiled-coil domain shared by all NMCP proteins. Together, these three changes are predicted to degrade the ability of the affected region to form a coiled-coil configuration (Supplementary Fig. S4B). These *nmcp2-3* homozygotes did not display striking whole-plant phenotypes; however, examination of leaf guard cells in these plants revealed changes in nuclear morphology. As illustrated in Fig. 6A, *nmcp2-3* guard cell nuclei were smaller (15 ± 4.1 µm^2^) than nuclei in wild-type guard cells (22 ± 5.0 µm^2^). In addition, nuclei in *nmcp2-3* guard cells were rounder than nuclei in wild-type guard cells, with a roundness index for *nmcp2-3* of 0.82 ± 0.10, compared with 0.68 ± 0.13 for the wild type. These nuclear morphology changes caused by the *nmcp2-3* allele closely matched the phenotypes caused by deficiencies in either *NMCP1A* or *NMCP1B*. This result demonstrates that NMCP2 participates with NMCP1-type proteins to specify or regulate nuclear morphology in tomato.

## Discussion

Using genome editing, we generated a collection of mutations disrupting or altering the function of the three *NMCP* genes encoding nuclear architectural proteins in cultivated tomato. We were interested in comparing the consequences of perturbing the nuclear lamina in Arabidopsis and tomato in light of the unique characteristics of Arabidopsis, which contains one of the smallest angiosperm genomes and has an abnormally low nuclear DNA-to-volume ratio (Fujimoto *et al.*, 2005). Accordingly, we hypothesized that a reduction in nuclear size resulting from nuclear lamina changes would lead to more severe phenotypes in tomato compared with Arabidopsis because the relatively crowded tomato nuclei would more quickly exceed a maximal DNA-to-volume threshold (Fujimoto *et al.*, 2005). Our data provide support for this hypothesis as developmental defects in *nmcp* mutants of tomato were more pronounced than those exhibited by comparable mutants in Arabidopsis (Dittmer *et al.*, 2007; Sakamoto and Takagi, 2013; Wang *et al*., 2013). These differences in developmental phenotypes will be discussed below, but first we will consider the similarities between *nmcp* mutants in tomato and Arabidopsis.

Overall, our work in tomato reinforces the importance of NMCP proteins in specifying, facilitating, or maintaining proper nuclear size and shape in flowering plants. Here, we show that tomato *nmcp* mutants have smaller and more spherically shaped nuclei within leaf guard cells. Examination of a second differentiated cell type, root epidermal cells, showed an interesting tissue specificity: only *nmcp1b* mutants displayed a shift in nuclear shape from elongated to more spherical. Similar changes in nuclear morphology occur in differentiated cells of Arabidopsis mutants carrying loss-of-function mutations in *NMCP* orthologs (i.e. *CRWN* genes) (Dittmer *et al.*, 2007; Sakamoto and Takagi, 2013; Wang *et al*., 2013). A recent study in maize indicates that a transposon-derived allele in the *MKAKU41* gene, which encodes a KAKU4-type nuclear lamina protein, also leads to less elongated nuclei in root hair cells (McKenna *et al.*, 2021). These results suggest that, at least in angiosperms (see below), NMCPs and an intact nuclear lamina allow nuclei to adopt non-spherical shapes.

The mechanisms through which NMCP mediates its effects on nuclear morphology remain unclear, but three general hypotheses can be advanced. The first possibility is that NMCPs are necessary for plasticity of the nuclear lamina or the nuclear margin. In this model, the reduced abundance or alteration of NMCP results in nuclei with a more rigid nuclear lamina that resists shape changes from physical forces exerted on the organelle within the plant cell, such as compression forces from the central vacuole (Obara *et al.*, 2001). Therefore, the *nmcp* mutant nuclei appear more spherical and uniform in shape, as opposed to the flattened, invaginated, and grooved forms of nuclei found in some wild-type plant cells (Collings *et al.*, 2000). This hypothesis inverts the expectations from work in animal cells, where lamin architectural proteins provide structural integrity to the nuclear lamina (Lammerding *et al.*, 2004; Swift *et al.*, 2013; Stephens *et al.*, 2018a, b) and prevent blebbing and structural malformations at the nuclear margin (Mounkes *et al.*, 2003; Vergnes *et al.*, 2004; Scaffidi and Misteli, 2006). Although NMCPs have been proposed to be lamin analogs (Ciska *et al.*, 2013; Meier *et al.*, 2017; Pradillo *et al.*, 2019), they differ structurally from their animal counterparts, lacking a globular Ig fold domain and a C-terminal prenylation motif (Gruenbaum and Foisner, 2015). Therefore, it is possible that NMCPs exert distinct effects on the properties of the nuclear lamina compared with animal lamins.

A second model posits that the crucial characteristic, in terms of nuclear morphology, that is impacted by NMCPs is the growth of the nuclear envelope. Overexpression studies in Arabidopsis have demonstrated that excess NMCP protein leads to a profusion of nuclear envelope growth (Sakamoto and Takagi, 2013; Goto *et al.*, 2014). Therefore, a reduction in NMCP abundance could lead to more spherical nuclei containing minimal nuclear sheaths stretched taut over the surface of their chromatin interiors. In this model, physical forces acting on the nuclear surface would have less purchase and less material with which to distort the envelope that defines the perimeter of the *nmcp* mutant nuclei.

A third possibility is that the absence of NMCPs disrupts connections between the nucleus and the cytoskeleton, as has been noted in mammalian cells deficient in lamin B1 (Ji *et al.*, 2007). Many of these connections are mediated by the LINC (LINKER of the NUCLEOSKELETON and CYTOPLASM) complex (Tatout *et al.*, 2014; Chang *et al.*, 2015). NMCP proteins contact integral inner nuclear membrane SUN-domain proteins (Graumann, 2014), which are core LINC components. Further, recent proximity ligation assay experiments identified microenvironments at the nuclear periphery in Arabidopsis containing NMCP proteins and LINC complex constituents (Tang *et al.*, 2020, 2022a, b). Therefore, it is possible that deficiencies in NMCPs could alter LINC abundance, distribution, or function in such a way as to change nuclear shape by abrogating cytoskeletal traction on the nuclear envelope.

Regardless of the mechanisms operating, we must consider the functional significance of distorted or elongated nuclear shapes in differentiated plant cells. The flexibility of nuclear shape might facilitate nuclear migration within cells, such as the bidirectional movement of nuclei in root hair cells (Chytilova *et al.*, 2000; Tamura *et al.*, 2013). This flexibility might also help nuclei withstand the shear forces applied by cytoplasmic streaming within plant cells (Shimmen and Yokota, 2004; Tominaga *et al.*, 2013). More generally, non-spherical shapes will increase the surface-to-volume ratio of the organelle and therefore might contribute to modulating the flux of nuclear import and export. Nonetheless, the lack of a strong phenotype in tomato *nmcp1a* or *nmcp2-3* mutants suggests that elongated nuclear shapes are not essential for the function of differentiated cells. While *nmcp1b* mutants in tomato do show significant phenotypes, their nuclear phenotype is not significantly different from that seen in *nmcp1a* or *nmcp2-3* mutants, pointing toward other explanations for the pleiotropy of *nmcp1b* mutations.

While important roles for NMCPs in nuclear cell biology are substantiated by genetic experiments in angiosperms, these roles might not extend to other plant groups. Obvious *NMCP* orthologs are absent in green algae (Poulet *et al.*, 2017), and a recent functional study from *Marchantia polymorpha*, a non-vascular model plant (a liverwort), showed that the single *NMCP* ortholog in this species can be mutated without significantly altering nuclear size or shape (Wang *et al*., 2021). However, loss of the unique NMCP ortholog in *Marchantia* is required for proper three-dimensional chromatin organization (Wang *et al*., 2021). These findings suggest that a role for NMCPs in organization of the nuclear genome through mediating contacts at the nuclear periphery (Wang *et al*., 2013; Hu *et al.*, 2019; Sakamoto *et al.*, 2020; Tang *et al.*, 2022a) might be conserved at the deepest nodes of the plant lineage. However, the *Marchantia* results also raise the intriguing possibility that the regulation of nuclear morphology by NMCPs might be limited to certain groups of plants.

In contrast to the situation in *Marchantia*, our previous results in Arabidopsis (Wang *et al*., 2013) and our new findings in tomato demonstrate that NMCP function is required for viability. In the case of tomato, both NMCP1-type and NMCP2-type genes are essential. In Arabidopsis, inactivation of the single NMCP2-type gene, *CRWN4*, leads to widespread gene expression and cytological changes, but the plants are viable without striking whole-plant phenotypes (Sakamoto and Takagi, 2013; Wang *et al*., 2013). In tomato, in contrast, *nmcp2* null mutations cause embryo lethality. A recent study by Masuda and colleagues showed that NMCP1- and NMCP2-type proteins form separate fibral networks in the nucleus of celery cells (Masuda *et al.*, 2021). The implication is that NMCP2 fibril networks, which are laterally associated with those formed by NMCP1-type proteins, are essential. If that is the case, why is CRWN4 dispensable in Arabidopsis? One possibility is that in Arabidopsis, as in other members of the *Brassicaceae*, there is a proliferation of a subgroup of NMCP1-type paralogs (Poulet *et al.*, 2017) (CRWN2 and CRWN3 in Arabidopsis; see Fig. 1). These proteins share the extended coiled-coil region at the boundary of their N-terminus with CRWN4 (Supplementary Fig. S4A), so it is possible that they may have some functional overlap with the single NMCP2-type protein in Arabidopsis. More work on the requirements of NMCP2 genes in other plant species will help clarify the situation. Recent work indicates that *nmcp2* mutants are viable in rice, as T-DNA insertion mutations in the single NMCP2-type protein-coding gene (*LOC_Os01g56140.1*) are not lethal and have an interesting drought hypersensitivity phenotype (Yang *et al.*, 2020). However, drawing a conclusion regarding this gene’s essential nature is complicated because the insertions in the two alleles recovered are in the 5ʹ region and in the final exon of the gene; therefore, these alleles might be leaky mutations.

Our genetic results in tomato add to our understanding of the functional significance of the N-terminus of NMCP proteins. This region is required in carrot for the subnuclear targeting to the nuclear rim (Kimura *et al.*, 2014). We were able to exploit natural variation in tomato to look at the consequences of deleting the entire N-terminal region in NMCP1A. In *nmcp1b* mutants, which display a number of developmental phenotypes, the plants are relying solely on an NMCP1-type protein lacking an N-terminal domain. Although the lower expression of the *NMCP1A* paralog compared with *NMCP1B* might be a factor, the severity of *nmcp1b* phenotypes, compared with those caused by *nmcp1a* null mutations, also suggests the possibility that NMCP1A minus the N-terminal domain is no longer able to carry out the full range of NMCP1-type functions.

The developmental phenotypes that result from reducing NMCP1-type function have been probed by varying the number of functional NMCP1-type gene alleles and taking advantage of the dosage sensitivity of *nmcp1a* alleles (with *1A*^*+/–*^*1B*^*–/–*^ showing more severe phenotypes than *1A*^*+/+*^*1B*^*–/–*^). These phenotypes include an unusual leaf asymmetry and folding phenotype that occurs with incomplete penetrance in both *nmcp1a* and *nmcp1b* mutants (see Supplementary Fig. S5). The affected leaves are typically limited to the terminal leaflet of the first emerging leaf, and hence this phenomenon is reminiscent of the ‘pointed first leaf’ mutants in Arabidopsis (Van Lijsebettens *et al.*, 1994; Ito *et al.*, 2000). The mutations responsible for that phenotype, and several subsequently isolated mutations that alter leaf symmetry in Arabidopsis (Degenhardt and Bonham-Smith, 2008; Pinon *et al.*, 2008; Yao *et al.*, 2008; Fujikura *et al.*, 2009; Horiguchi *et al.*, 2011), affect ribosomal proteins and probably translation rates. How nuclear lamina mutations might fit into this framework is unclear, but it is possible that altering nuclear shape could elicit leaf morphology aberrations by a similar mechanism if the export of rRNA and ribosome biogenesis decrease.

Perhaps the most striking developmental phenotypes caused by the reduction in NMCP1-type function is slow root growth. As shown in Fig. 2C, loss of NMCP1B function leads to a dramatic reduction in the growth rate of the primary root. Reducing NMCP1-type function further in a *1A*^*+/–*^*1B*^*–/–*^ mutant leads to an arrest in growth of the primary root that is compensated by the formation and subsequent growth of secondary rootlets (Fig. 5B). The basis of this root growth phenotype remains to be determined, but a root growth phenotype has also been observed in the rice mutants carrying T-DNA insertions in the NMCP2-type gene, *LOC_*Os01g56140, linked to a reduction in the size of the root meristematic zone (Yang *et al.*, 2020). We have seen dwarfing in Arabidopsis individuals carrying multiple *crwn* mutations (Wang *et al*., 2013), a consequence of the ectopic induction of stress response pathways (Guo *et al.*, 2017; Choi *et al.*, 2019; Jarad *et al.*, 2019; Wang *et al*., 2019). In tomato, however, *nmcp1* mutants do not exhibit lesion-mimic phenotypes, which are indicative of the ectopic pathogen signaling that is a hallmark of Arabidopsis *crwn1 crwn2* double mutants (Choi *et al.*, 2019). The non-overlapping spectrum of whole-plant phenotypes seen in tomato compared with Arabidopsis *nmcp* mutants suggests that the nuclear lamina coordinates or influences distinct transcriptional and signaling pathways in the two species.

## Supplementary data

The following supplementary data are available at *JXB* online.

Fig. S1. A haplotype network of the first exon of orthologous *NMCP1A* genes in related *Solanum* species.

Fig. S2. Gene expression patterns of the three tomato *NMCP* genes.

Fig. S3. CRISPR/Cas9-targeted mutagenesis of the three *NMCP* genes in tomato.

Fig. S4. An extended coiled-coil domain in NMCP2-type proteins and the predicted effects of the *nmcp2-3* mutation.

Fig. S5. Leaflet folding in *nmcp1a* and *nmcp1b* mutants.

Fig. S6. Loss of NMCP1B, but not NMCP1A, affects nuclear morphology in root epidermal cells.

Fig. S7. Leaf and stature phenotypes of *1A*^*+/–*^*1B*^*–/–*^ plants.

Table S1. Oligonucleotide primers used in this study.

Table S2. Nucleotide sequences used to construct the haplotype network in Supplementary Fig. S1.

erad294_suppl_Supplementary_Figures_S1-S7_Tables_S1-S2

## Data Availability

The data are available in the article and in its supplementary data.
