# Supplementary material for: The nuclear lamina is required for proper development and nuclear shape distortion in tomato
Source: J Exp Bot. 2023 Jul 28;74(18):5500–13. doi: 10.1093/jxb/erad294 (PMC10540737; doi:10.1093/jxb/erad294)
Supplement: erad294_suppl_Supplementary_Figures_S1-S7_Tables_S1-S2 [file erad294_suppl_supplementary_figures_s1-s7_tables_s1-s2.pdf]

SUPPLEMENTARY DATA

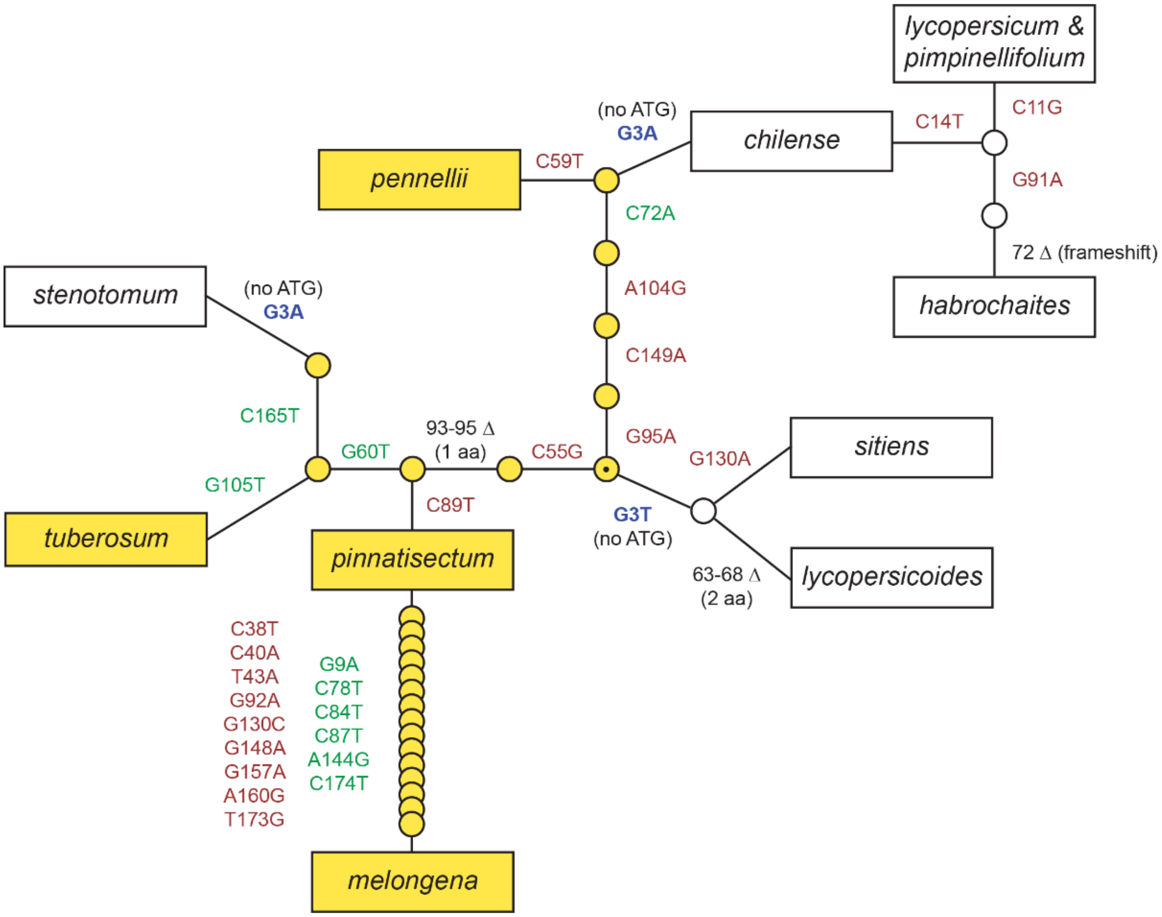

Supplementary Fig. S1. A haplotype network of the first exon of orthologous *NMCP1A* genes in related *Solanum* species. Ten haplotypes corresponding to eleven species are shown in the rectangles; haplotypes marked in yellow encode an N-terminal domain while haplotypes in open boxes do not encode an N-terminal domain. Deduced intermediary haplotypes are shown as small circles with a similar color coding. A haplotype toward the center the network was chosen as a reference (dotted circle) and mutational steps connecting the haplotypes are shown with the nucleotide position indicated. The three independent polymorphisms coded in blue inactivate the initiator codon of the ORF encoding the N-terminal domain. Other polymorphisms leading to non-synonymous substitutions are shown in red; green polymorphisms are silent in terms of amino acid coding. Deletions are denoted by triangles; these deletions lead to either an amino acid deletion (in two cases) or a frameshift in the ORF coding the N-terminal domain. A total of 15 nucleotide changes connect *S. melongena* (eggplant) to the rest of the network.

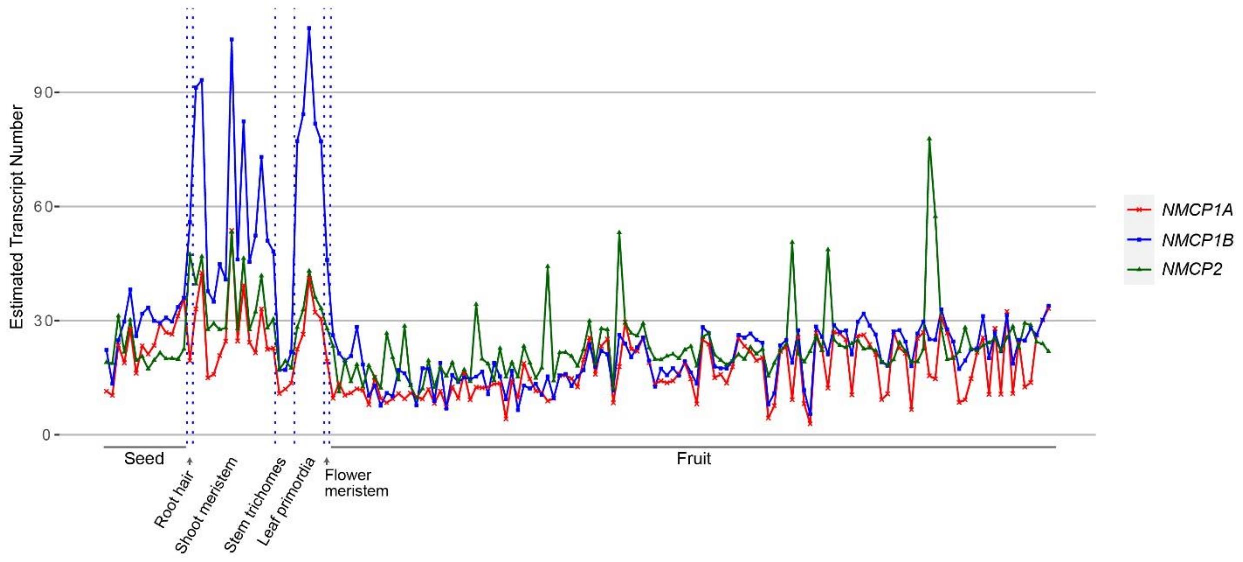

Supplementary Fig. S2. Gene expression patterns of the three tomato *NMCP* genes. The graph represents RNA-seq data organized in the TomExpress database (v21.0) (https://tomexpress.gbfwebtools.fr/) (Zouine *et al.*, 2017). A total of 159 conditions (tissue and developmental time points) were extracted for the three tomato *NMCP* genes; all samples were from M82 strain. Tissue sources are represented on the x-axis. For most tissues, multiple samples were taken over a developmental time course, progressing left to right.

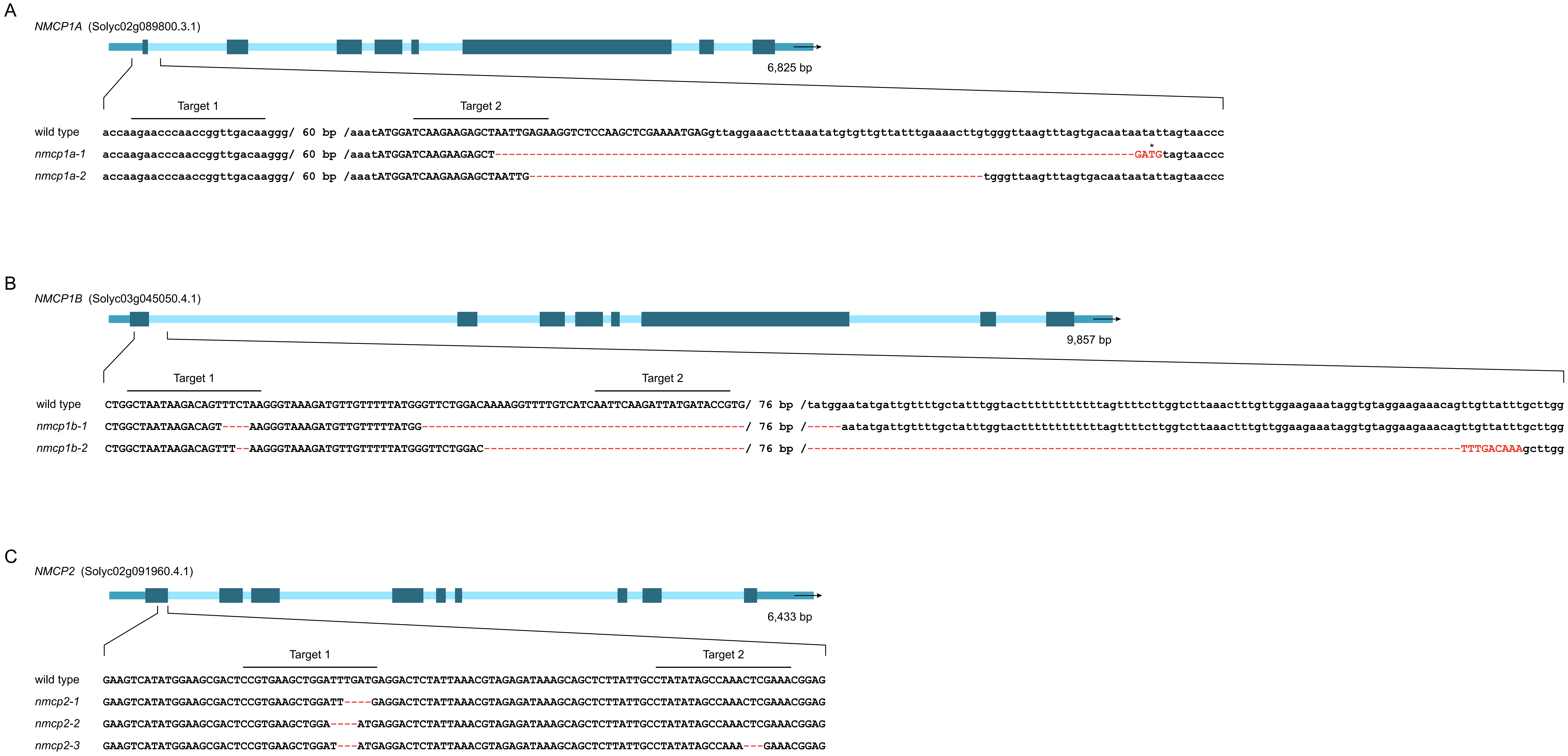
A
NMCP1A (Solyc02g089800.3.1)
6,825 bp
Target 1
Target 2
wild type accaagaacccaaccggttgacaaggg/ 60 bp /aaatATGGATCAAGAAGAGCTAATTGAGAAGGTCTCCAAGCTCGAAAATGAGgttaggaaactttaaatatgtgttgttatttgaaaacttgtgggttaagtttagtgacaataatattagtaaccc
nmcp1a-1 accaagaacccaaccggttgacaaggg/ 60 bp /aaatATGGATCAAGAAGAGCT------GATGtagtaaccc
nmcp1a-2 accaagaacccaaccggttgacaaggg/ 60 bp /aaatATGGATCAAGAAGAGCTAATTG------tgggttaagtttagtgacaataatattagtaaccc
B
NMCP1B (Solyc03g045050.4.1)
9,857 bp
Target 1
Target 2
wild type CTGGCTAATAAGACAGTTTCTAAGGGTAAAGATGTTGTTTTTATGGGTTCTGGACAAAAGGTTTTGTCATCAATTCAAGATTATGATACCGTG/ 76 bp /tatggaatatgattgttttgctatttggtactttttttttttttagttttcttggtcttaaactttgttggaagaaataggtgtaggaagaaacagttgttatttgcttgg
nmcp1b-1 CTGGCTAATAAGACAGT----AAGGGTAAAGATGTTGTTTTTATGG------/ 76 bp /-----aatatgattgttttgctatttggtactttttttttttttagttttcttggtcttaaactttgttggaagaaataggtgtaggaagaaacagttgttatttgcttgg
nmcp1b-2 CTGGCTAATAAGACAGTTT--AAGGGTAAAGATGTTGTTTTTATGGGTTCTGGAC------/ 76 bp /------TTTGACAAAgcttgg
C
NMCP2 (Solyc02g091960.4.1)
6,433 bp
Target 1
Target 2
wild type GAAGTCATATGGAAGCGACTCCGTGAAGCTGGATTTGATGAGGACTCTATTAAACGTAGAGATAAAGCAGCTCTTATTGCCTATATAGCCAAACTCGAAACGGAG
nmcp2-1 GAAGTCATATGGAAGCGACTCCGTGAAGCTGGATT----GAGGACTCTATTAAACGTAGAGATAAAGCAGCTCTTATTGCCTATATAGCCAAACTCGAAACGGAG
nmcp2-2 GAAGTCATATGGAAGCGACTCCGTGAAGCTGGA----ATGAGGACTCTATTAAACGTAGAGATAAAGCAGCTCTTATTGCCTATATAGCCAAACTCGAAACGGAG
nmcp2-3 GAAGTCATATGGAAGCGACTCCGTGAAGCTGGAT---ATGAGGACTCTATTAAACGTAGAGATAAAGCAGCTCTTATTGCCTATATAGCCAAA---GAAACGGAG

Supplementary Fig. S3. CRISPR/Cas9-targeted mutagenesis of the three *NMCP* genes in tomato. The intron/exon structure of (A) *NMCP1A*, (B) *NMCP1B*, and (C) *NMCP2* are shown at the top of each panel. Coding exons are depicted as thicker darker blue boxes with pale blue introns and untranslated regions (5'- and 3'-UTRs) in an intermediate intensity blue. The arrow signifies the direction of transcription. Magnified regions containing the sequences within the first exon targeted by the two guide RNAs (Target 1 and Target 2) are shown below their respective gene models. The top nucleotide sequence corresponds to the wild-type sequence of the gene in cultivar M82, with uppercase letters indicating coding exon sequences and lowercase letters used for introns or 5'-UTRs. The nucleotide sequences of the recovered engineered alleles are aligned below the wild-type sequence: deletions are denoted as red dashes; insertions are shown as red nucleotides. The asterisk in panel A indicates the position of the splice donor used in transcripts from the *nmcp1a-1* allele. Table 1 summarizes the alterations in each allele.

A

```
NMCP1A     ---------------------------------------------------------------------------------M 1
NMCP1B     MSTPPRKVFSGWTLTPRTDLANK---TVSKGKDVVFMGSGQKVLSSI-------QDYDTV---------------------- 50
CRWN1      -MSTPLKVWQRWSTPTKATNPDSNGSSHGTGLDMVTPVSGRV--S-E-------IQFD------------------------ 47
NMCP2      -MASPGSGRLALTPVN---------------------PTPISGLGRVSKTPLT-------DEVIWKRLREAGFDEDS-IKRR 52
CRWN4      MATSSRSERFPITPSTAATN-----------------RLTITPNSRVLKSPLT-------EEIMWKRLKDAGFDEQS-IKNR 57
CRWN2      --MTPRSETHKIGGVTNPRNADRKGKAVAFSDDLVIPTLPPPPIGTLTGQGVSRGHTDDMDMGDWRRFREVGLLNEASMEKK 80
CRWN3      -MFTPQ--------RNRWPETDRKGKAIAFSDEIITPPP----QRVLLR-----------EDDDWQKFKEVGLLDEASLERK 58
                                                                       :   *:::::.*: ::: ::.:
```

Extended CC

```
NMCP1A     DQEELIEKVSKLENELFDYQYNMGLILLEKKEWSSKFEEIKQTLEESNEAYRREQAAHLIAISEVEKREENLRKALGVEKQF 83
NMCP1B     DKVVLLDKVSKLENELVDYQYNMGLLLIEKKEWSAKLEEIKQALSEANEAYRREHTAHLIALSEVEKREENLRKALGVENQC 132
CRWN1      DPRILPEKISELEKELFEYQHSMGLLLIEKKEWSSQYEALQQAFEEVNECLKQERNAHLIAIADVEKREEGLRKALGIEKQC 129
NMCP2      DKAALIAYIAKLETELYDHQYQMGLLILERKEWVSKNEQSKAASESAELLYKREQAARLSDTAEAKKLEANLKKALGIEKEC 134
CRWN4      DKAALIAYIAKLESEVYDYQHNMGLLLLEKNELSSQYEEIKASVDESDLTHMREKSAYVSALAEAKKREESLKKDVGIAKEC 139
CRWN2      DQEALLEKISTLEKELYGYQHNMGLLLMENKELVSKHEQLNQAFQEAQEILKREQSSHLYALTTVEQREENLRKALGLEKQC 162
CRWN3      DRDALIEKILKLEKELFDYQHNMGLLLIEKKQWTSTNNELQQAYDEAMEMLKREKTSNAITLNEADKREENLRKALIDEKQF 140
           *   *   :  **.*:  :*:.***:::*.::  :  :  : : ..      :*: :       ..: * .*:* :   ::
```

Conserved Coiled Coil

B

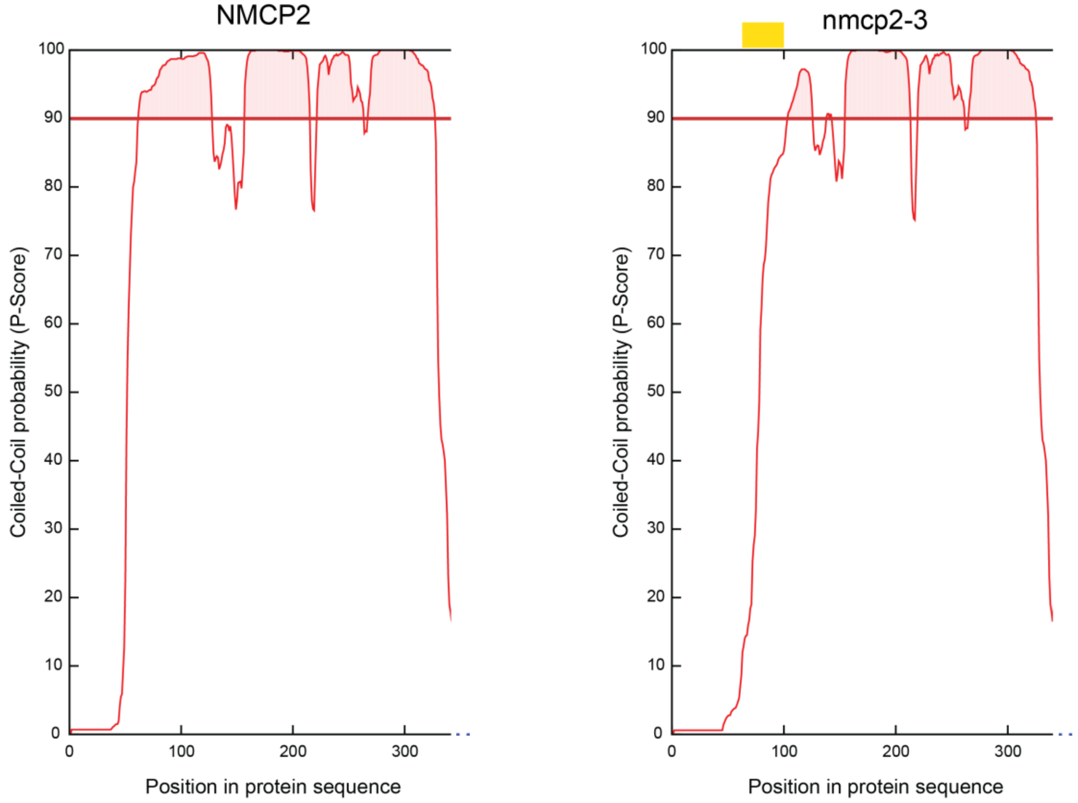

Supplementary Fig. S4. An extended coiled-coil domain in NMCP2-type proteins and the predicted effects of the *nmcp2-3* mutation. (A) Alignment of the N-terminal region of NMCPs in Arabidopsis and tomato; residue coordinates are shown on the right of the stacked amino acid sequence alignment. Gaps have been added, denoted as dashes, to maximize alignment. The position of coiled-coil regions predicted by Multicoil2 (Simm *et al.*, 2015) are shown below the sequence. Note the extended coiled-coil region shared by the NMCP2-type proteins in the two species (tomato NMCP2 and Arabidopsis CRWN4). This region is also shared by two NMCP1-type proteins from Arabidopsis: CRWN2 and CRWN3. The amino acids highlighted in yellow are either deleted (red amino acid residues) or altered (green amino acid residue) in the protein encoded by the tomato *nmcp2-3* allele (also, see Table 1). (B) A comparison of predicted coiled-coil regions in the first one-third of the wild type NMCP2 protein type (left panel, NMCP2) and the protein encoded by the *nmcp2-3* allele (right panel, nmcp2-3). The yellow rectangle on the top right panel highlights the region in the mutant protein that is predicted to no longer form a coiled-coil.

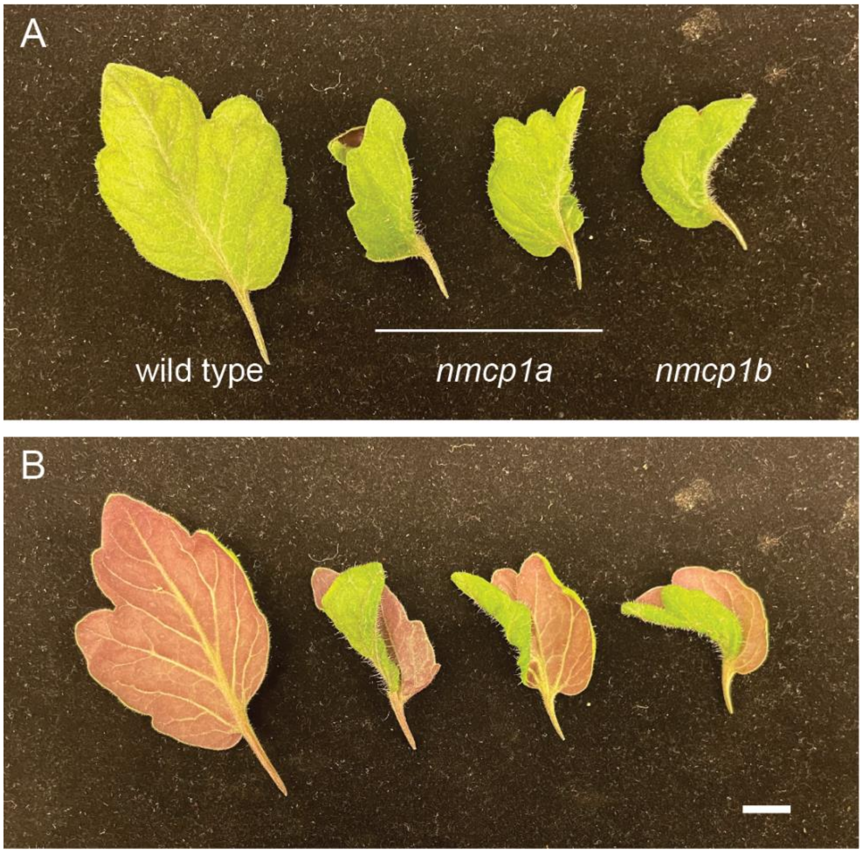

Supplementary Fig. S5. Leaflet folding in *nmcp1a* and *nmcp1b* mutants. (A) The adaxial surface of terminal leaflets of the first or second emerging leaves of *nmcp* mutant plants and that of a wild-type control. Plants were grown in parallel and are 4-weeks-old. In this cohort, terminal leaflet folding occurred in 0 of 5 wild-type control plants; 2 of 8 *nmcp1a* mutant plants; and 3 of 5 *nmcp1b* mutant plants. (B) Abaxial surfaces of the same leaflets shown in (A) highlight the sharp folding of the blade, typically along the mid-vein, which is often asymmetrically positioned relative to the whole blade surface. Bar, 1 cm.

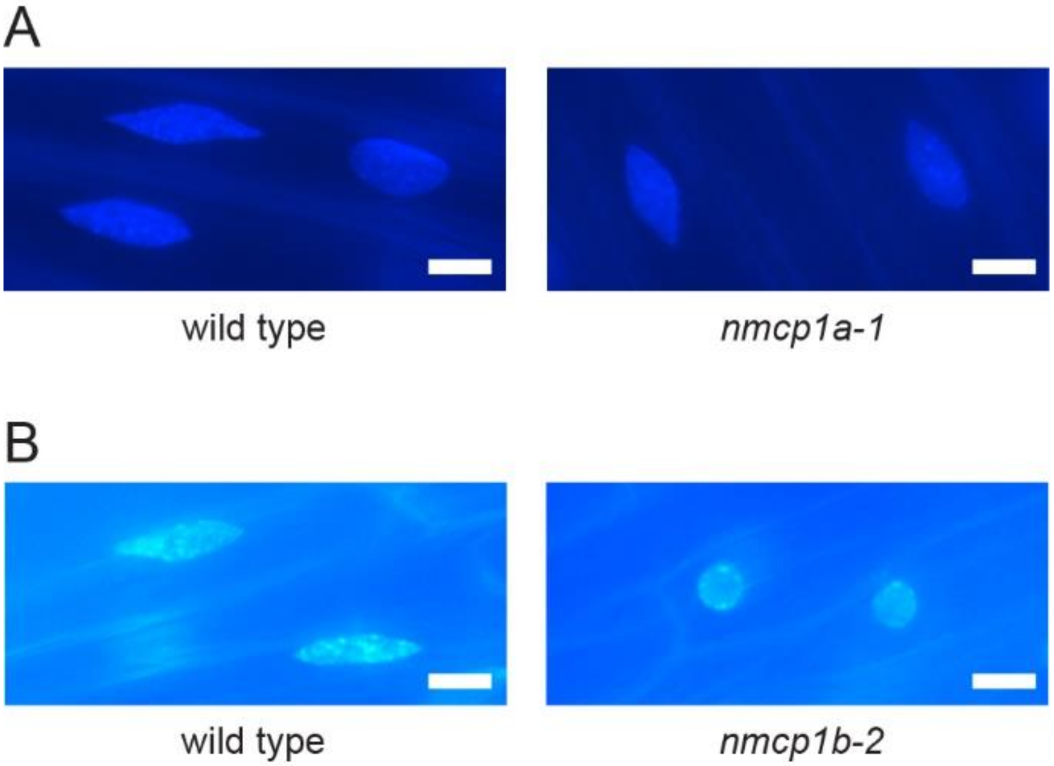

Supplementary Fig. S6. Loss of NMCP1B, but not NMCP1A, affects nuclear morphology in root epidermal cells. A comparison of representative DAPI-stained nuclei from fixed root epidermal tissue derived from seedlings; matched wild-type controls are shown on the left and either *nmcp1a* (A) or *nmcp1b* (B) mutant is shown on the right. Bar, 10 µm.

A B

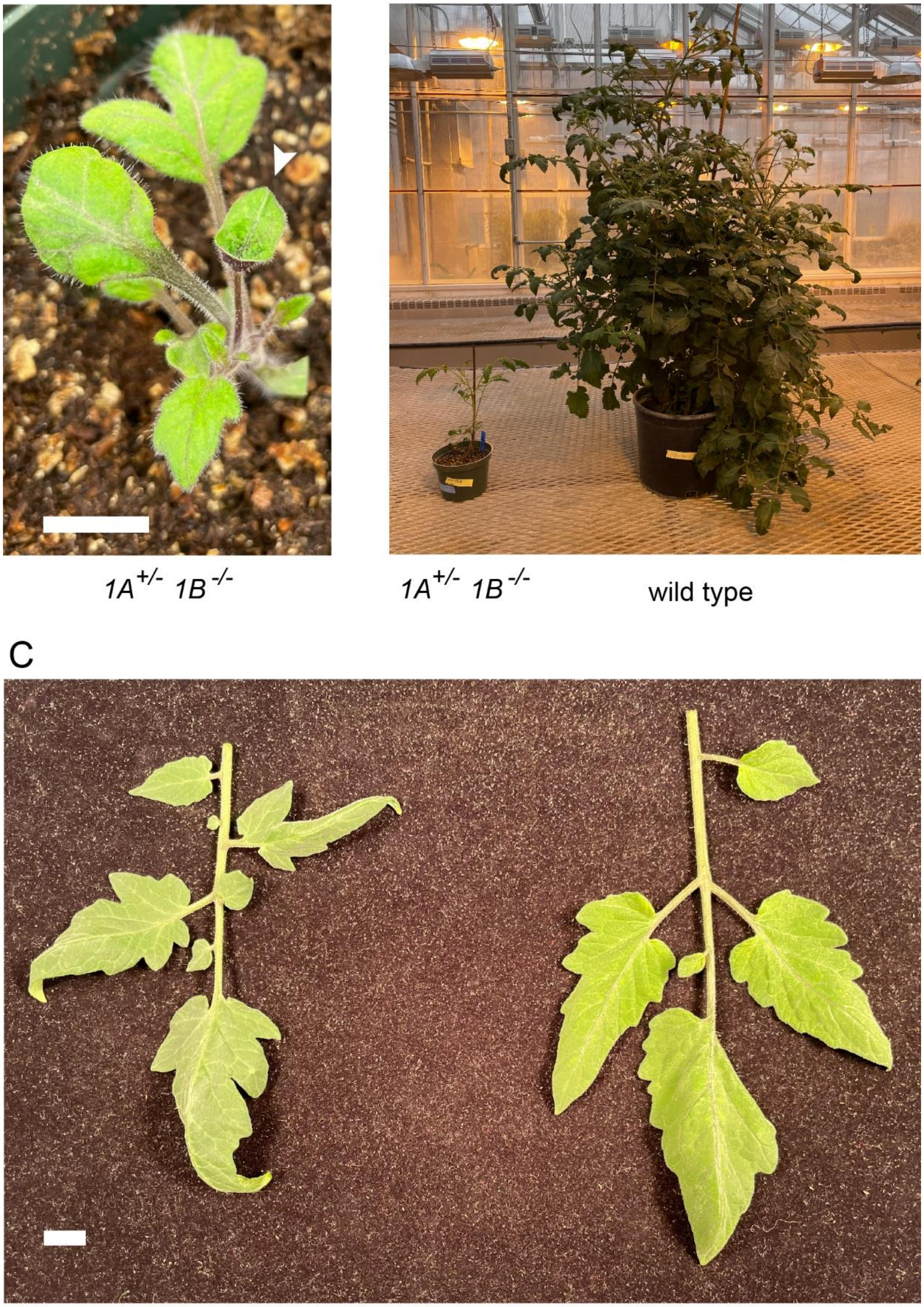

1A$^{+/-}$ *1B*$^{-/-}$

1A$^{+/-}$ *1B*$^{-/-}$ wild type

C

1A$^{+/-}$ *1B*$^{-/-}$ wild type

Supplementary Fig. S7. Leaf and stature phenotypes of *1A[+/-] 1B[-/-]* plants. (A) A four-week-old *1A[+/-] 1B[-/-]* individual with a trumpet-shaped leaf (arrowhead). Bar, 1 cm. (B) Two 13-week-old plant in the greenhouse showing the size disparity between a wild-type control plant (right) and a different *1A[+/-] 1B[-/-]* individual (left). For a size reference, the *1A[+/-] 1B[-/-]* individual on the left is in a '6-inch azalea' pot (diameter of the top rim is approximately 15 cm). (C) Terminal leaflets of an adult leaf from a *1A[+/-] 1B[-/-]* individual (left) compared to a set of terminal leaflets from a developmentally-matched leaf from a wild type individual (right). Bar, 1 cm.

Supplementary Table S1. Oligonucleotide primers used in this study

Oligonucleotide name	Sequence (5' to 3')	Purpose
Cas9-pr99	ACGAGGTGGCGTACCATG	Cas9 genotyping, primer 1
Cas9-pr100	TTCCTCCTGGCTTGCTCC	Cas9 genotyping, primer 2
SlNMCP1A.1-pr103	GTTGTCAATCTGTGATTCCTG	*NMCP1A* genotyping, primer 1
SlNMCP1A.1-pr104	ACATCAGCTTCACATACGAG	*NMCP1A* genotyping, primer 2
SlNMCP1B-pr115	GTCTACCCCGCCGAGAAA	*NMCP1B* genotyping, primer 1
SlNMCP1B-pr116	ACAGAACAAGCACAGCCTAC	*NMCP1B* genotyping, primer 2
SlNMCP2-pr107	AGTCCTGGGTCAGGGAG	*NMCP2* genotyping, primer 1
SlNMCP2-pr108	TGGCGTCTACTAAGAACTGAAC	*NMCP2* genotyping, primer 2
NMCP1A-F	CGGAAATATGGATCAAGAAGAG	*NMCP1A* cDNA synthesis, primer 1
NMCP1A-R	CTCGTGCAAATTGCTTC	*NMCP1A* cDNA synthesis, primer 2
NMCP1A-1-T1	tgtggtctcaATTGAGAACCCAACCGGTTGACAAgttttagagctagaaatagcaag	gRNA forward primer for NMCP1A, target 1
NMCP1A-1-T2	tgtggtctcaATTGTCAAGAAGAGCTAATTGAGAgttttagagctagaaatagcaag	gRNA forward primer for NMCP1A, target 2
NMCP1B-T1	tgtggtctcaATTGCTAATAAGACAGTTTCTAAgttttagagctagaaatagcaag	gRNA forward primer for NMCP1B, target 1
NMCP1B-T2	tgtggtctcaATTGAATTCAAGATTATGATACCGgttttagagctagaaatagcaag	gRNA forward primer for NMCP1B, target 2
NMCP2-T1	tgtggtctcaATTGCCGTGAAGCTGGATTTGATGgttttagagctagaaatagcaag	gRNA forward primer for NMCP2, target 1
NMCP2-T2	tgtggtctcaATTGCTATATAGCCAAACTCGAAAgttttagagctagaaatagcaag	gRNA forward primer for NMCP2, target 2
CRISPR-Rev	tgtggtctcaAGCGTAATGCCAACTTTGTAC	gRNA reverse primer

Supplementary Table S2. Nucleotide sequences used to construct the haplotype network in Fig. S1

Solanum species (strain)	Nucleotide sequence	GenBank Accession or Reference
chilense (LA3111)	ATATCTACGCCGCCGAGGAAATCTTGGACCGGGTGGTCGCTGTCACCAAGAACCCAACCGGTTG<br>ACAAGGGAAAAGGCATCGCCTTCACGGGTGATACTGCTCGGAAAAGTTTGACGAGCCAAGATTA<br>CGGAAATATGGATCAAGAAGAGCTAATTGAGAAGGTCTCCAAGCTCGAAAATGAG	(Stam *et al.*, 2019)
habrochaites (LA1777)	ATATCTACGCCGCTGAGGAAATCTTGGACCGGGTGGTCGCTGTCACCAAGAACCCAACCGGTTG<br>ACAAGGGAAAGGCATCGCCTTCACGAGTGATACTGCTCGGAAAAGTTTGACGAGCCAAGATTAC<br>GGAAATATGGATCAAGAAGAGCTAATTGAGAAGGTCTCCAAGCTCGAAAATGAG	Susan Strickler, personal communication
lycopersicoides (LA2951)	ATTTCTACGCCGCCGAGGAAATCTTGGACCGGGTGGTCGCTGTCACCAAGAACCCAACCGGTGG<br>GCAAAGGCATCGCCTTCACGGGTGGTACTGCTCAGAAAAGTTTGACGAGCCAAGATTACGGAAA<br>TATGGATCAAGAAGCGCTAATTGAGAAGGTCTCCAAGCTCGAAAATGAG	(Powell *et al.*, 2022)
lycopersicum	ATATCTACGCGGCTGAGGAAATCTTGGACCGGGTGGTCGCTGTCACCAAGAACCCAACCGGTTG<br>ACAAGGGAAAAGGCATCGCCTTCACGGGTGATACTGCTCGGAAAAGTTTGACGAGCCAAGATTA<br>CGGAAATATGGATCAAGAAGAGCTAATTGAGAAGGTCTCCAAGCTCGAAAATGAG	XM_004232049.4
melongena ('67/3')	ATGTCTACACCGCCGAGGAAATCTTGGACCGGGTGGTTGATGACACCAAGAACCGAACCGGTTG<br>ACAAGGGCAAAGGTATCGCTTTTATGGATACTGCTCAGAAAAGTTTGACGAGCCAAGATTACCG<br>AAATATGGATCAGGAAACGCTAATTAAGGAGGTCTCCAAGCGTGAAAATGAG	(Barchi *et al.*, 2021)
pennellii	ATGTCTACGCCGCCGAGGAAATCTTGGACCGGGTGGTCGCTGTCACCAAGAACCCAACTGGTTG<br>ACAAGGGAAAAGGCATCGCCTTCACGGGTGATACTGCTCGGAAAAGTTTGACGAGCCAAGATTA<br>CGGAAATATGGATCAAGAAGAGCTAATTGAGAAGGTCTCCAAGCTCGAAAATGAG	XM_015210708.2
pimpinellifolium (LA1670)	ATATCTACGCGGCTGAGGAAATCTTGGACCGGGTGGTCGCTGTCACCAAGAACCCAACCGGTTG<br>ACAAGGGAAAAGGCATCGCCTTCACGGGTGATACTGCTCGGAAAAGTTTGACGAGCCAAGATTA<br>CGGAAATATGGATCAAGAAGAGCTAATTGAGAAGGTCTCCAAGCTCGAAAATGAG	(Takei *et al.*, 2021)
pinnatisectum	ATGTCTACGCCGCCGAGGAAATCTTGGACCGGGTGGTCGCTGTCACCAAGAACCGAACCGGTTG<br>ACAAGGGCAAAGGCATCGCCTTCATGGGTACTGCTCAGAAAAGTTTGACGAGCCAAGATTACGG<br>AAATATGGATCAAGAAGCGCTAATTGAGAAGGTCTCCAAGCTCGAAAATGAG	CP047559.1
sitiens (LA1974)	ATTTCTACGCCGCCGAGGAAATCTTGGACCGGGTGGTCGCTGTCACCAAGAACCCAACCGGTTG<br>ACAAGGGCAAAGGCATCGCCTTCACGGGTGGTACTGCTCAGAAAAGTTTGACGAGCCAAGATTA<br>CAGAAATATGGATCAAGAAGCGCTAATTGAGAAGGTCTCCAAGCTCGAAAATGAG	(Molitor *et al.*, 2021)
stenotomum	ATATCTACGCCGCCGAGGAAATCTTGGACCGGGTGGTCGCTGTCACCAAGAACCGAACCTGTTG<br>ACAAGGGCAAAGGCATCGCCTTCACGGGTACTGCTCAGAAAAGTTTGACGAGCCAAGATTACGG<br>AAATATGGATCAAGAAGCGCTAATTGAGAAGGTTTCCAAGCTCGAAAATGAG	XM_049528885.1
tuberosum	ATGTCTACGCCGCCGAGGAAATCTTGGACCGGGTGGTCGCTGTCACCAAGAACCGAACCTGTTG<br>ACAAGGGCAAAGGCATCGCCTTCACGGGTACTGCTCATAAAAGTTTGACGAGCCAAGATTACGG<br>AAATATGGATCAAGAAGCGCTAATTGAGAAGGTCTCCAAGCTCGAAAATGAG	XM_006338219.2

REFERENCES

Barchi L, Rabanus-Wallace MT, Prohens J, Toppino L, Padmarasu S, Portis E, Rotino GL, Stein N, Lanteri S, Giuliano G. 2021. Improved genome assembly and pan-genome provide key insights into eggplant domestication and breeding. Plant J **107**, 579-596.

Molitor C, Kurowski TJ, Fidalgo de Almeida PM, Eerolla P, Spindlow DJ, Kashyap SP, Singh B, Prasanna H, Thompson AJ, Mohareb FR. 2021. *De novo* genome assembly of *Solanum sitiens* reveals structural variation associated with drought and salinity tolerance. Bioinformatics **37**, 1941-1945.

Powell AF, Feder A, Li J, Schmidt MH, Courtney L, Alseekh S, Jobson EM, Vogel A, Xu Y, Lyon D, Dumschott K, McHale M, Sulpice R, Bao K, Lal R, Duhan A, Hallab A, Denton AK, Bolger ME, Fernie AR, Hind SR, Mueller LA, Martin GB, Fei Z, Martin C, Giovannoni JJ, Strickler SR, Usadel B. 2022. A *Solanum lycopersicoides* reference genome facilitates insights into tomato specialized metabolism and immunity. Plant J **110**, 1791-1810.

Simm D, Hatje K, Kollmar M. 2015. Waggawagga: comparative visualization of coiled-coil predictions and detection of stable single alpha-helices (SAH domains). Bioinformatics **31**, 767-769.

Stam R, Nosenko T, Horger AC, Stephan W, Seidel M, Kuhn JMM, Haberer G, Tellier A. 2019. The *de novo* reference genome and transcriptome assemblies of the wild tomato species *Solanum chilense* highlights birth and death of NLR genes between tomato species. G3 (Bethesda) **9**, 3933-3941.

Takei H, Shirasawa K, Kuwabara K, Toyoda A, Matsuzawa Y, Iioka S, Ariizumi T. 2021. *De novo* genome assembly of two tomato ancestors, *Solanum pimpinellifolium* and *Solanum lycopersicum* var. cerasiforme, by long-read sequencing. DNA Res **28**.

Zouine M, Maza E, Djari A, Lauvernier M, Frasse P, Smouni A, Pirrello J, Bouzayen M. 2017. TomExpress, a unified tomato RNA-seq platform for visualization of expression data, clustering and correlation networks. Plant J **92**, 727-735.
